# Identification of Stages of Erythroid Differentiation in Bone Marrow and Erythrocyte Subpopulations in Blood Circulation that Are Preferentially Lost in Autoimmune Hemolytic Anemia in Mouse

**DOI:** 10.1371/journal.pone.0166878

**Published:** 2016-11-21

**Authors:** Sreoshi Chatterjee, Nitin Bhardwaj, Rajiv K. Saxena

**Affiliations:** 1 School of Life Sciences, Jawaharlal Nehru University, New Delhi, India; 2 Faculty of Life Sciences and Biotechnology, South Asian University, New Delhi, India; CCAC, UNITED STATES

## Abstract

Repeated weekly injections of rat erythrocytes produced autoimmune hemolytic anemia (AIHA) in C57BL/6 mice after 5–6 weeks. Using the double *in vivo* biotinylation (DIB) technique, recently developed in our laboratory, turnover of erythrocyte cohorts of different age groups during AIHA was monitored. Results indicate a significant decline in the proportion of reticulocytes, young and intermediate age groups of erythrocytes, but a significant increase in the proportion of old erythrocytes in blood circulation. Binding of the autoantibody was relatively higher to the young erythrocytes and higher levels of intracellular reactive oxygen species (ROS) were also seen in these cells. Erythropoietic activity in the bone marrows and the spleen of AIHA induced mice was examined by monitoring the relative proportion of erythroid cells at various stages of differentiation in these organs. Cells at different stages of differentiation were enumerated flow cytometrically by double staining with anti-Ter119 and anti-transferrin receptor (CD71) monoclonal antibodies. Erythroid cells in bone marrow declined significantly in AIHA induced mice, erythroblast C being most affected (50% decline). Erythroblast C also recorded high intracellular ROS level along with increased levels of membrane-bound autoantibody. No such decline was observed in spleen. A model of AIHA has been proposed indicating that binding of autoantibodies may not be a sufficient condition for destruction of erythroid cells in bone marrow and in blood circulation. Last stage of erythropoietic differentiation in bone marrow and early stages of erythrocytes in blood circulation are specifically susceptible to removal in AIHA.

## Introduction

Autoimmune hemolytic anemia (AIHA) is one of the earliest recognized autoimmune diseases in humans [1], characterized by the production of self-reactive autoantibodies against erythrocytes that can lead to a rapid and profound decline of erythrocyte count and hemoglobin concentration in blood [2–5]. Pathogenesis of AIHA involves two underlying mechanisms, *viz*, erythrophagocytosis of autoantibody-coated erythrocytes by macrophages in the reticulo-endothelial system in liver and spleen [6,7], and complement mediated lysis of erythrocytes following binding of IgM autoantibodies [8]. AIHA has been extensively investigated mainly for the understanding of its pathogenesis, clinical features and prognosis [3,6,9,10]. However the modulation of erythropoietic homeostasis and the turnover pattern of circulating erythrocytes are poorly understood. It is also not known if the age of erythrocytes in circulation has a bearing on their susceptibility to elimination in mice with AIHA.

Erythroid line of differentiation in bone marrow and spleen starts with the early progenitor pro-erythroblasts that are derived from the pluripotent stem cells. Pro-erythroblasts further differentiate in successive stages *viz* erythroblast A, B and C [11,12]. These four stages of erythroid differentiation in bone marrow and spleen can be enumerated by flow cytometric analysis of bone marrow and spleen cells stained with Ter-119 and CD71 antibodies. Erythroid cells are released from bone marrow and spleen as reticulocytes that rapidly (within 1 to 2 days) differentiate into mature erythrocytes in blood [11,12]. Average half-life of blood erythrocytes in mice is about 60 days. It has been difficult to enumerate and study at any given time point the proportions of erythrocytes of various age groups in blood circulation. A new technique of double in vivo biotinylation (DIB technique) of erythrocytes developed recently in our laboratory has however made it possible to simultaneously enumerate and study erythrocytes of different age groups in blood circulation [13–19]. Aim of the present study was to look at the complete life cycle of erythroid cells including different stages of differentiation in bone marrow and spleen as well as erythrocytes of different age groups in blood circulation in order to identify stages that are preferentially destroyed in autoimmune hemolytic anemia. In order to accomplish this objective, we studied changes that occur in mice with AIHA in (a) relative proportions of cells in different stages of erythroid differentiation in bone marrow and spleen, as well as the relative proportions of reticulocytes and erythrocytes of different age groups in blood circulation, and (b) the binding of autoantibodies, and the generation of reactive oxygen species (ROS) in cells in different stages of erythroid life cycle in bone marrow, spleen and blood. Our results indicate that while autoantibodies bind to cells in all stages of erythropoiesis in bone marrow and spleen and circulating mature erythrocytes, decline in relative proportion was confined only to the late stages of erythroid differentiation in bone marrow and younger erythrocytes in blood circulation suggesting that these erythroid populations may be preferentially lost in AIHA.

## Materials and Methods

### Animals

Inbred C57BL/6 male mice (8–12 weeks old, 20–25 g body weight) and female Wistar rats (2 months old, 250–300 g body weight) were used throughout this study. Animals were bred and maintained in microbe free environment in the animal house facility at Jawaharlal Nehru University (JNU), New Delhi or obtained from the National Institute of Nutrition (NIN), Hyderabad. The animals were housed in positive-pressure air conditioned units (25°C, 50% relative humidity) and kept on a 12 h light/dark cycle. Water and mouse chow were provided *ad libitum*. All the experimental protocols were conducted strictly in compliance with the Standard Operating Procedures (SOP) for Institutional Animal Ethics Committee (IAEC) of the CPCSEA (Committee for the Purpose of Control and Supervision on Experiments on Animals), Ministry of Environment, Forest and Climate Change, Government of India (http://www.moef.nic.in/sites/default/files/SOP_CPCSEA_inner_page%20%281%29.pdf). The study was duly approved by Institutional Animal Ethics Committee of Jawaharlal Nehru University (IAEC Approved Project Code: 35/2012). All mice were randomly assigned to experimental groups. Experiments were designed so as to use the minimum number of mice.

For the analysis of blood erythrocytes, 20–25 μl blood samples were taken weekly from tail-vein at indicated time points. For deriving bone marrow and spleen cells, mice were euthanized by CO_2_ asphyxiation before the organs were dissected out.

### Reagents and other Supplies

Biotin-X-NHS (N-hydroxysuccinimide ester of biotin) was obtained from Sigma Aldrich (St. Louis, MO, USA). Streptavidin-Allophycocyanin (SAv-APC), rat anti-mouse Ter-119-APC, rat anti-mouse CD71-Phycoerythrin (PE), rat anti-mouse CD71-Fluorescein isothiocyanate (FITC) monoclonal antibodies, anti-mouse CD16/CD32 purified, goat anti-mouse IgG/IgM-FITC polyclonal antibody, and Annexin V-PE and Annexin V-FITC recombinant proteins were purchased from BD Biosciences (San Diego, CA, USA) or from Affymetrix eBioscience (San Diego, CA, USA). Goat F(ab’)_2_ anti-mouse IgG-PE polyclonal antibody, rat IgG1κ-PE, rat IgG1κ-FITC, rat IgG2aκ-PE and rat IgG2bκ-APC isotype controls, and 7-Aminoactinomycin D (7AAD) were procured from Affymetrix eBioscience (San Diego, CA, USA). 5 (and 6)—chloromethyl-2,7-dichloro-dihydrofluorescein diacetate (CM-H_2_DCFDA) was purchased from Molecular Probes (Eugene, OR, USA). Fetal bovine serum (FBS) was obtained from Hyclone (South Logan, UT, USA). RPMI was obtained from Sigma-Aldrich (St. Louis, MO, USA). HEPES, Dimethylformamide (DMF), Dimethyl sulfoxide (DMSO), Ethylene diamine tetra acetic acid (EDTA) and other analytical reagents were from Sigma-Aldrich (India). Mounting medium, Fluoromount G was purchased from G Biosciences (St. Louis, MO, USA). All other chemicals were purchased locally and were of analytical grade.

### Induction of autoimmune hemolytic anemia in mice

Experimental autoimmune hemolytic anemia (AIHA) was induced in mice following the Playfair and Clarke model of repeated injections with rat erythrocytes [20–23]. Rat RBCs derived from tail vein were washed 3 times in phosphate-buffered saline (PBS, pH 7·4) and adjusted to a concentration of 1x10^9^ cells/ml. Mice were given weekly injections of 2x10^8^ rat RBCs intraperitoneally. Blood samples (20–25 μl) from mice were collected in PBS containing 5 mM EDTA, at different time points from the tail vein. Erythrocyte count and hemoglobin levels were estimated by using an electronic hematology particle counter (MS4Se, Melet Schloesing Laboratories, Chaussée Jules César, Osny, France). Membrane-bound autoantibodies were detected by staining the erythrocytes with anti-mouse IgG/IgM-FITC or F(ab’)_2_-anti-mouse IgG-PE followed by flow cytometry [24,25].

### Double *in vivo* biotinylation (DIB) technique

Double *in vivo* biotinylation (DIB) of erythrocytes was done as described previously [13–19]. The DIB technique involves two steps of biotinylation of circulating erythrocytes by intravenous (*i*.*v*.) administration of biotin-X-NHS Ester (BXN), through the tail vein of mice. In the first step of high intensity *in vivo* biotinylation, three daily *i*.*v*. injections of biotin (1 mg BXN dissolved in 20 μl of DMF and 250 μl of PBS) were given, followed after 30 days, by a low intensity biotinylation with a single lower dose (0.6 mg of BXN dissolved in 12 μl of DMF and 250 μl of PBS). This low intensity biotinylation labels the fresh erythrocytes that were released in circulation in the 30-day period following the first biotinylation step. At any time point after the second biotinylation step, biotin intensity on circulating erythrocytes could be analyzed by flow cytometry using streptavidin coupled to an appropriate fluorochrome, as described before [13–15]. Biotin^negative^ erythrocytes in circulation would represent fresh and youngest erythrocytes released in blood after the second biotinylation step. Biotin^low^ erythrocytes would represent the cohort of erythrocytes released in circulation between the first and the second biotinylation steps, and biotin^high^ erythrocytes would represent the population of old residual erythrocytes that were present in blood at the time of first biotinylation step [18]. The schedule of biotinylation was fixed so that at the intended time of analysis (i.e., after 5–6 injections) the circulating erythrocytes comprise a very young cohort of biotin^negative^ erythrocytes (less than 6 or 13 days old, depending upon the day of sacrifice) and a very old cohort of biotin^high^ erythrocytes (more than 36 or 43 days old, depending upon the day of sacrifice) along with an intermediate aged biotin^low^ erythrocyte cohort. The principle of DIB technique is summarized in S1 Fig.

### Erythroid differentiation in bone marrow and spleen

For deriving bone marrow and spleen cells, mice were euthanized by CO_2_ asphyxiation before the organs were dissected out. Bone marrow (BM) cells were flushed out of femur and tibia using a 25-gauze needle and resuspended in RPMI medium with 10% FBS. Single cell suspensions of spleen cells were made by gently teasing the spleen in a small volume of PBS. Splenic and BM cells were strained through a fine sieve, pelleted by centrifugation, and resuspended at desired concentration in RPMI with 10% FBS. For delineating erythroid precursors at different stages of differentiation, freshly prepared single cell suspensions from BM or spleen were first incubated with anti-CD16/32 monoclonal antibody (Fc block, 1 μg/10^6^ cells in 50 μl of PBS + 2% FBS) for 10 mins followed by staining with anti-mouse CD71-PE/FITC and anti-mouse Ter-119-APC for 20 mins at 4°C [19,26–27].

### Measurement of intracellular Reactive Oxygen Species (ROS)

Intercellular reactive oxygen species (ROS) level was assessed as described before [19, 28–30]. Briefly, erythrocytes from peripheral blood and primary cells from BM and spleen were washed and resuspended in pre-warmed PBS supplemented with 2% FBS and incubated with CM-H_2_DCFDA (5(and 6-)-chloromethyl-2,7-dichloro-dihydrofluorescein diacetate) stain (5 μM) in the dark for 30 minutes at 37°C in an atmosphere of 5% CO_2_ in air. The oxidative conversion of CM-H_2_DCFDA to its fluorescent product by ROS was measured immediately by flow cytometry [19,28,30]. Intracellular ROS generation in different subpopulations of erythrocytes and erythroid precursor cells were determined by gating the cells on the basis of biotin label and CD71 stain, or Ter119 and CD71 stain, respectively. ROS fluorescence signals (MFI) were recorded in these gated populations.

### Flow Cytometric Analysis

Mouse blood was collected in PBS containing 5 mM EDTA and washed 3 times with ice cold saline containing HEPES buffer (10 mM, pH-7.4) and 2% FBS. Biotin-labeled erythrocytes (1×10^6^) were stained *ex vivo* with streptavidin-APC and anti-mouse CD71-PE/FITC in dark for 30 minutes to identify the different age cohorts of erythrocytes, as described before [13,18]. To enumerate the level of membrane-bound autoantibody in different subpopulations of circulating erythrocytes, cells (1×10^6^) were co-stained with anti-mouse IgG/IgM-FITC along with streptavidin-APC and anti-mouse CD71-PE. To determine other markers these DIB labeled erythrocytes were stained with the appropriate antibodies/dyes and their corresponding expression was assessed in the erythrocyte subpopulations by gating the cells on the basis of biotin label and CD71 stain.

For enumerating erythroid cells at different stages of differentiation in BM and spleen the technique of double staining with anti-mouse CD71 and anti-mouse Ter119 was used, as described before [19,26,27]. Briefly freshly prepared single cell suspensions (1×10^6^) from BM or spleen were incubated with anti-mouse CD16/CD32 antibody (Fc block, 1 μg/10^6^ cells in 50 μl of PBS + 2% FBS) for 10 mins followed by staining with anti-mouse CD71-PE and anti-mouse Ter-119-APC for 20 min in dark at 4°C. To determine other markers, these erythroid cells were stained with the appropriate antibodies/dyes and their corresponding expression was assessed in the erythroid precursors by gating the cells on the basis of CD71 and Ter119 staining. To detect autoantibodies bound to erythroid cells, rat serum was used for blocking instead of mouse serum, and cells were stained with F(ab’)_2_ anti-mouse IgG-PE together with anti-mouse CD71-FITC and anti-mouse Ter-119-APC for 20 mins in dark at 4°C.

All the stained cells were washed and analyzed immediately on a flow cytometer. For all the flow cytometric analysis 7AAD was used as viability dye and immunophenotyping was carried out on gated live 7AAD^-ve^ cells. A minimum of 10,000 events were recorded for erythrocytes and 50,000 events for analyzing erythroid subpopulations in BM and spleen. All the flow cytometric analyses were performed on a BD FACSCalibur flow cytometer (Becton Dickinson, San Jose, CA, USA) using Cell Quest software, or on a BD FACSVerse flow cytometer (Becton Dickinson, San Jose, CA, USA) using FACSuite software for acquisition and analysis.

### Fluorescent Microscopy

Mouse blood was collected in PBS containing 5 mM EDTA and washed 3 times with ice cold saline containing HEPES buffer (10 mM, pH-7.4) and 2% FBS. Freshly isolated erythrocytes were stained with goat F(ab’)_2_ anti-mouse IgG-PE monoclonal antibody in dark for 30 mins and fixed with 4% Paraformaldehyde (PFA) in PBS containing 1mM EGTA and 2.5 mM MgCl_2_ for 30 min at room temperature. Fixed erythrocytes were layered on Poly L-Lysine (PLL) coated cover slips, and allowed to adhere for 30 min in dark. After incubation the cover slips containing erythrocytes were washed in PBS and mounted onto slides using mounting medium (Fluoromount) and stored at 4°C in dark until analyzed. Images were collected on a confocal laser scanning microscope Olympus FLUOVIEW FV1000 using the Olympus FLUOVIEW software (Ver.1.7a).

### Statistical Analysis

Each experiment was repeated at least three times. Statistical analysis by Student’s t-test and ANOVA was carried out using SigmaPlot software. Data are presented as means ± SEM. A level of *p*<0.05 was accepted as statistically significant.

## Results

### Induction of Autoimmune hemolytic anemia in mice

**Fig 1:**
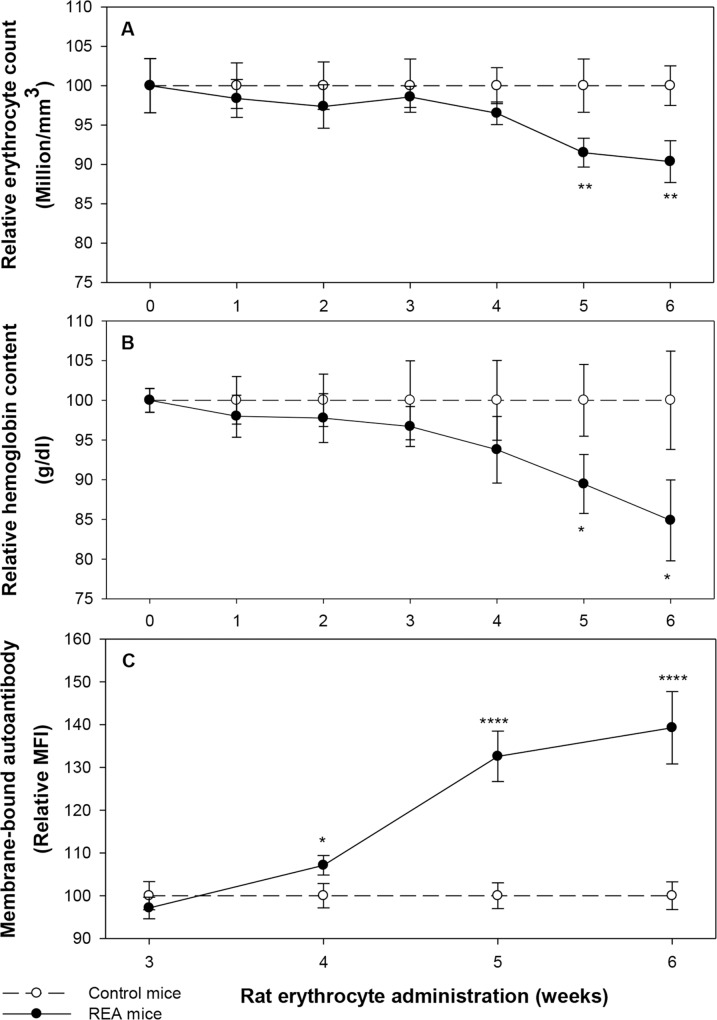
Induction of autoimmune hemolytic anemia (AIHA) in mice. Mice were injected weekly 2x10^8^ rat erythrocytes intraperitoneally. Blood samples from mice in control and rat-erythrocyte-administered (REA) mice were collected at different time points and analyzed on an automated cell counter. Relative changes in the erythrocyte count and hemoglobin content in the REA mice over a period of 6 weeks are given in panels A and B respectively (*p* = 0.001 for erythrocytes, and *p =* 0.013 for hemoglobin after 5–6 injections, ANOVA). Presence of anti-mouse erythrocyte autoantibody was estimated by staining the erythrocytes with anti-mouse IgG/IgM-FITC following flow cytometry. Relative binding of autoantibodies (MFI) to erythrocytes over a period of 4 weeks, from 3^rd^-6^th^ week, (*p<*0.001, ANOVA) is given in panel C. Each point on the graph represents mean ± SEM of observations. n = 10 control and 15 REA mice. *p<0.05, **p<0.01 and ****p<0.001 for comparison of the groups (Student t-test).

AIHA was induced in mice by multiple administrations of rat erythrocytes. Mice were administered (*i*.*p*.) rat erythrocytes weekly and blood erythrocyte count and hemoglobin levels were monitored in control and rat-erythrocyte-administered (REA) mice. At each time point, the mean blood erythrocyte count in control mice was taken as hundred and the relative changes in the blood erythrocyte count in REA mice were determined. The results shown in Fig 1, panels A and B indicate that a significant anemia was demonstrable in REA mice after 5 weekly administrations of rat erythrocytes. A 10% fall in blood erythrocyte count (p<0.001) was noted at 5 and 6 week time points (Fig 1, panel A). Similarly, a significant decline in blood hemoglobin level was noted in REA mice after 5 weekly administrations of rat erythrocytes, decline being about 15% (p = 0.013) on 6 week time point (Fig 1, panel B). Presence of erythrocyte-bound autoantibody on blood erythrocytes in REA mice was revealed by staining the blood derived erythrocytes with FITC conjugated anti mouse Ig polyclonal antibody, followed by flow cytometric analysis (S2 Fig). A sharp increase (up to 40%, p<0.001) in the mean fluorescence intensity (MFI) of bound autoantibody was seen in erythrocytes of REA mice at 5 and 6 week time-points (Fig 1, panel C).

**Fig 2:**
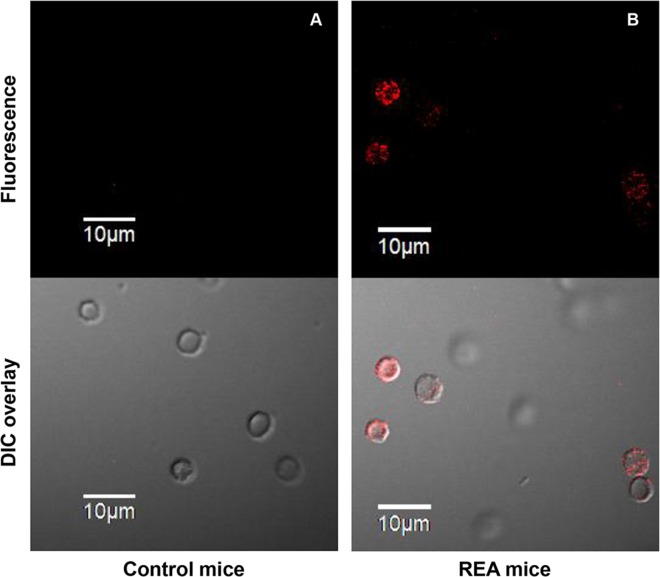
Fluorescence imaging of erythrocytes showing the presence of membrane-bound autoantibodies. Mice were injected weekly with 2x10^8^ rat erythrocytes intraperitoneally for 5 weeks. Blood samples from mice in control and REA groups were collected after 5 injections. Presence of membrane-bound autoantibodies in erythrocytes isolated from REA mice was confirmed by fluorescence microscopy after staining the erythrocytes with F(ab’)_2_ anti-mouse IgG-PE. Panel A shows the erythrocyte images from control and panel B the same from REA mice. The upper panels display fluorescence images and lower panels the DIC overlay (magnification 100X).

Presence of membrane-bound autoantibodies on erythrocytes from REA mice was further confirmed by fluorescence microscopy. Erythrocytes from REA mice were stained with F(ab’)_2_ anti-mouse IgG-PE polyclonal antibody and visualized on a fluorescence microscope. Erythrocytes from REA mice clearly show the presence of membrane-bound autoantibodies tagged with PE-conjugated secondary antibody (Fluorescence images Fig 2 upper panel, DIC overlay Fig 2 lower panel).

### Turnover of erythrocytes in mice with AIHA

**Fig 3:**
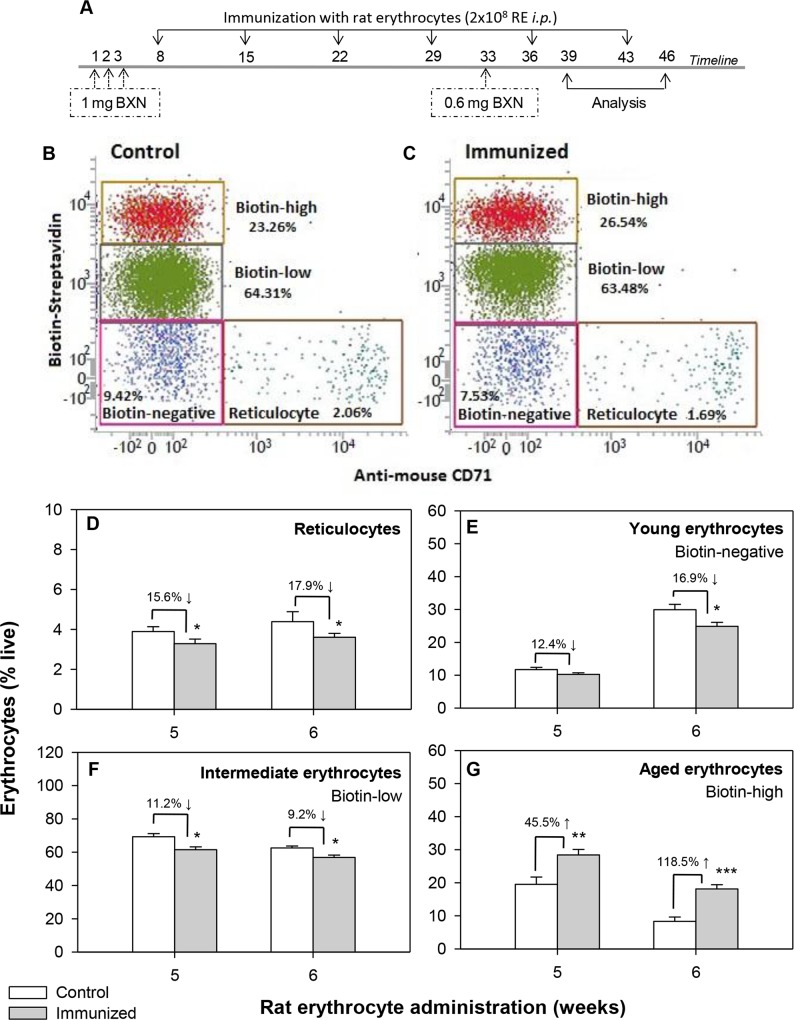
Erythrocyte turnover in the blood of control and AIHA-induced mice. Mouse erythrocytes were labeled with biotin *in vivo* by the two step biotinylation procedure. DIB labeled mice were given intraperitoneal injections of 2x10^8^ rat erythrocytes weekly for 5–6 weeks to induce AIHA. The experimental protocol is given in panel A. Blood samples were collected from control and AIHA-induced mice 3 days after the 5^th^ and 6^th^ injections (6 and 13 days after 2^nd^ biotinylation step). Erythrocytes were stained *ex vivo* with streptavidin-APC and anti-mouse CD71-PE, and proportions of the different age cohorts were determined. Representative flow histograms showing the proportion of different age groups of erythrocytes after 5 doses of injections are shown in panels B (control) and C (AIHA-induced). Turnover profile of reticulocytes (panel D; *p* = 0.02, ANOVA), biotin^negative^ young erythrocytes (panel E; *p* = 0.046, ANOVA), biotin^low^ intermediate age group of erythrocytes (panel F; *p* = 0.010, ANOVA) and biotin^high^ old erythrocytes (panels G; *p*<0.001, ANOVA) in control and AIHA-induced mice have been shown. Each bar on the graph represents mean ± SEM of observations. n = 8 control and 12 AIHA-induced mice. *p<0.05, **p<0.01 and ***p<0.005 for comparison of the groups (Student t-test).

To assess whether the decline in blood erythrocyte count in AIHA mice was due to a uniform loss of erythrocytes across all age groups or whether younger or older erythrocytes were preferentially lost in AIHA, the DIB technique of erythrocyte labeling was employed [13,18] (concept explained in S1 Fig). Experimental protocol showing the schedule of rat erythrocyte administration along with the biotinylation steps and the time points for collecting blood samples is shown in Fig 3, panel A. The schedule of the two biotinylation steps was planned so that at the time of analysis (*i*.*e*., after the onset of AIHA as a result of 5 weekly *i*.*p*. injections of rat erythrocytes), three distinct subgroups of very young biotin^negative^ (<6 days old), intermediate age erythrocytes (biotin^low^ 6 to 36 days old), and very old biotin^high^ (>36 days old) erythrocytes could be identified. Young erythrocyte group could further be subdivided into reticulocytes and young erythrocytes based upon staining with CD71 antibody. Erythrocytes isolated from the peripheral blood were stained *ex vivo* with streptavidin-APC and anti-mouse CD71-PE, and proportions of biotin^high^, biotin^low^, CD71^˗^ biotin^negative^ and CD71^+^biotin^negative^ populations were determined by flow cytometry. Panels B and C of Fig 3 show representative results of the relative proportions of reticulocytes, biotin^negative^, biotin^low^ and biotin^high^ populations of erythrocytes in control and AIHA-induced mice respectively. As compared to control, a relatively lower proportion of biotin^negative^ (young) erythrocytes and high proportion of biotin^high^ (old) erythrocyte population in AIHA-induced mice was observed in these representative results.

Combined data from 8 control and 12 AIHA-induced mice for proportion of different age groups of erythrocytes and reticulocytes in peripheral blood, are shown in Fig 3, panels D-G. These results indicate a significant decline in the proportion of reticulocytes and younger erythrocytes in mice after 6 weekly-injections of rat erythrocytes (Fig 3, panels D-E). The intermediate age group of erythrocytes (biotin^low^ erythrocytes that enter the blood circulation within a window of 30 days between the first and second biotinylation steps) also follow the same pattern as the young biotin^negative^ erythrocytes (Fig 3, panel F). The older erythrocyte population (biotin^high^) however showed a significant increase in AIHA-induced mice (Fig 3, panel G). Proportion of young (<6days old) and intermediate (6–36 days old) erythrocytes dropped from 11.69 ± 0.70% and 69.22 ± 1.91% in control to 10.25 ± 0.48 and 56.80 ± 1.46% (*p =* 0.010, ANOVA test) respectively in the AIHA-induced mice. The proportion of aged (>43 days old) erythrocytes increased 2-folds from 8.31 ± 1.34% in control to 18.15 ± 1.27% (*p<*0.001, ANOVA) in the mice with induced AIHA. These results suggest that relatively younger erythrocytes in blood may be preferentially eliminated in conditions of AIHA. The 16–18% decline in reticulocyte proportion (4.39 ± 0.50% in control to 3.60 ± 0.20% in mice after 6 injections) could further be an indication of depressed erythropoietic activity in AIHA-induced mice (Fig 3, panel D).

### Levels of membrane bound autoantibodies on reticulocytes and erythrocytes of different ages in AIHA mice

**Fig 4:**
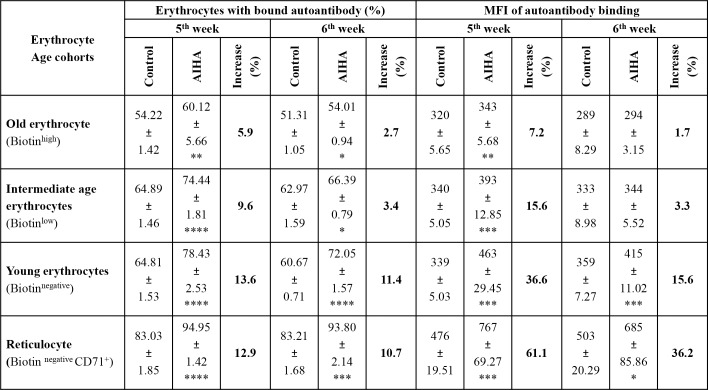
Autoantibody binding in erythrocytes of different age groups. DIB labeled mice were given weekly *i*.*p*. injections of 2x10^8^ rat erythrocytes for 5–6 weeks to induce AIHA. Blood samples were collected from control and AIHA mice 3 days after the 5^th^ and 6^th^ injection. Erythrocytes were stained *ex vivo* with anti-mouse IgG-PE, streptavidin-APC and anti-mouse CD71-FITC. Erythrocytes of different age groups were gated and autoantibody level was analyzed in each of them. Each value represents mean ± SEM of data. n = 8 control and 12 AIHA mice. *p<0.05, **p<0.01, ***p<0.005 and ****p<0.001 for comparison of the groups (Student t-test). ANOVA test for autoantibody binding on different age groups of erythrocytes significant (*p* = 0.001 in terms of MFI and *p* = 0.021 in terms of erythrocyte proportion with membrane-bound autoantibody).

Levels of membrane bound anti-erythrocyte autoantibody were estimated on erythrocytes of different age groups as well as on reticulocytes. Erythrocytes from mice treated with DIB protocol were stained with streptavidin-APC and anti-mouse CD71-PE to demarcate reticulocytes and erythrocyte of different age groups, and co-stained with anti-mouse IgG/IgM-FITC polyclonal antibodies to reveal the membrane bound autoantibodies. Populations of reticulocytes and erythrocytes of different age group were gated on flow cytometer on the basis of streptavidin vs. CD71 plots and presence of membrane bound autoantibodies analyzed on all these populations. Results in Fig 4 show a significant increase in autoantibody binding in all age groups of erythrocytes including reticulocytes. Binding of anti-mouse erythrocyte autoantibody showed a significant rise in erythrocytes from AIHA mice both in terms of proportion of erythrocytes with membrane-bound autoantibodies and mean fluorescence intensity (MFI) of bound autoantibody. Further, maximum binding of the autoantibody was seen in reticulocytes and young erythrocytes and there was a clear decline in autoantibody binding in erythrocytes of intermediate and old age.

### Age dependent changes in levels of ROS in erythrocytes from mice with AIHA

**Fig 5:**
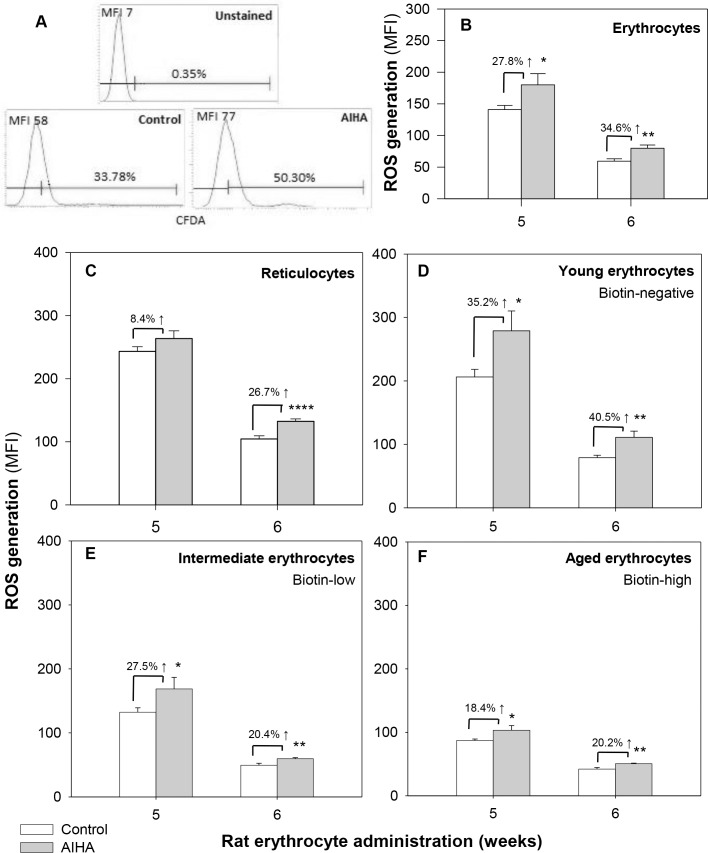
Generation of Reactive Oxygen Species (ROS) in control and AIHA-induced mice. Mice were given intraperitoneal injections of 2x10^8^ rat erythrocytes weekly for 5–6 weeks to induce AIHA. Blood samples were collected from control and AIHA-induced mice 3 days after 5^th^ and 6^th^ doses of injection. Erythrocytes were stained with CM-H_2_DCFDA and intracellular ROS generation was determined by flow cytometry. Representative histograms showing ROS generation in control and AIHA-induced mice are shown in panel A, and the mean ROS level in whole erythrocyte population is shown in panel B (*p* = 0.010, ANOVA). Mouse erythrocytes were labeled with biotin *in vivo* by the two step biotinylation procedure (as per schedule given in Fig 3, panel A). Erythrocytes from DIB stained mice were incubated with CM-H_2_DCFDA and stained *ex vivo* with streptavidin-APC and anti-mouse CD71-PE. Erythrocytes of different age groups (reticulocytes, biotin^negative^, biotin^low^ and biotin^high^) were gated (as in Fig 3, panels B and C) and ROS level was analyzed in each of them. ROS level in the different age cohorts of erythrocytes are given in panels C-F. ANOVA tests for each of the subgroups reveal *p =* 0.741 for reticulocytes (panel C), *p* = 0.009 for biotin^negative^ (panel D), *p* = 0.022 for biotin^low^ (panel E) and *p =* 0.005 for biotin^high^ (panel F). Each bar on the graph represents mean ± SEM of observations. n = 8 control and 12 AIHA-induced mice. *p<0.05, **p<0.01 and ****p<0.001 for comparison of the groups (Student t-test).

Oxidative damage has been implicated in the pathogenesis of a number of autoimmune disorders [31–35]. We therefore examined ROS generation in the erythrocytes in mice with experimentally induced AIHA. ROS levels in circulating erythrocytes in control and AIHA-induced mice were estimated by staining with CM-H_2_DCFDA [19,28,30]. Results indicate a significant increase in the MFI of ROS fluorescence in the whole blood erythrocyte population in AIHA-induced mice (Fig 5 panels A and B). After 6 injections of rat erythrocytes, the ROS level in AIHA-induced mice was 79.80 ± 5.15 as compared to 59.29 ± 3.93 in control, a 1.3-fold increase (*p =* 0.010, ANOVA).

ROS generation was also examined separately in reticulocytes and blood erythrocyte cohorts of different ages (reticulocytes, biotin^negative^, biotin^low^ and biotin^high^) in DIB labeled mice. Results in Fig 5, panels C-F show that similar to the pattern observed for the membrane bound autoantibody, ROS generation was also significantly higher in all the age groups of erythrocytes, the effect being maximal in the younger biotin^negative^ erythrocytes. After 6 injections, the ROS generation in biotin^negative^ erythrocytes increased to 111.00 ± 9.85 from 79.00 ± 3.86 in control (Fig 5, panel D), a 1.4-fold increase, followed by a 30% increase in reticulocytes whose MFI increased to 132.44 ± 3.70 from 104.50 ± 4.87 (Fig 5, panel C). ROS levels were relatively lower in intermediate and old age groups of erythrocytes. These results suggest that younger subpopulation of erythrocytes may generate more ROS in mice with AIHA.

### Erythropoietic activity in AIHA-induced mice

Bone marrow and spleen, the two prime erythropoietic sites in adult mice, were examined for alterations in erythropoietic pattern as a consequence of induction of AIHA. Differential expression of Ter119 and CD71 molecules on erythroid cell surface in BM and spleen were used to delineate four distinct stages of erythroid differentiation: early pro-erythroblasts (Ter119^med^ CD71^high^), early basophilic erythroblasts (Ter119^high^ CD71^high^ FSC^high^, erythroblast A), late basophilic, polychromatic and orthochromatic erythroblasts (Ter119^high^ CD71^med^ FSC^low^, erythroblast B) and orthochromatic erythroblasts with mature erythrocytes (Ter119^high^ CD71^low^ FSC^low^, erythroblast C), as described elsewhere [19,26,27]. Representative flow histograms showing the gating strategies for identifying different stages of erythroid differentiation in mice BM is shown in S3 Fig.

**Fig 6:**
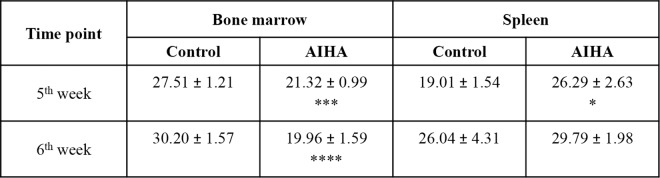
Total erythroid cells in control and AIHA induced mice. Mice were given *i*.*p*. injections of 2x10^8^ rat erythrocytes weekly for 5–6 weeks to induce AIHA. Mice were sacrificed 3 days after 5^th^ and 6^th^ doses of injection and their BM and spleen cells were harvested. Cells isolated were stained with anti-mouse CD71-PE, anti-mouse Ter119-APC and 7AAD, after blocking with anti-mouse CD16/32, and the proportions of erythroid cells (as described in S3 Fig) were determined. Proportions of erythroid cells in BM and spleen of mice are given above (BM *p*<0.001 and spleen *p* = 0.061, ANOVA). Each value represents mean ± SEM of observations. n = 4 control and 6 AIHA mice. *p<0.05, ***p<0.005 and ****p<0.001 for comparison of the groups (Student t-test).

**Fig 7:**
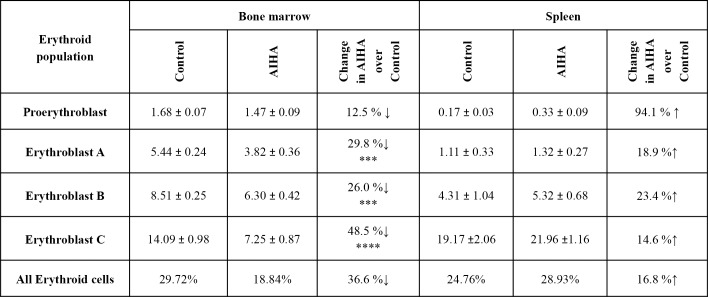
Changes in relative proportions of erythroid cells in different stages of differentiation in AIHA. Mice were given weekly *i*.*p*. injections of rat erythrocytes to induce AIHA. Mice were sacrificed 3 days after 6^th^ injection and their BM and spleen cells were harvested. Cells isolated were stained with anti-mouse CD71-PE, anti-mouse Ter119-APC and 7AAD, and proportion of erythroid cells were determined as described in S3 Fig. Proportion of erythroid cells in different maturational stages of development in BM and spleen in control and AIHA mice are given along with percent changes in AIHA (ANOVA, *p*<0.001 for both BM and spleen). Each value represents mean ± SEM of observations. n = 4 control and 6 AIHA-induced mice. *p<0.05, ***p<0.005 and ****p<0.001 for comparison of the groups (Student t-test).

Proportions of all erythroid cells (Ter119 positive cells) in bone marrow and spleens of control and AIHA mice are shown in Fig 6. Proportion of erythroid cells in bone marrow (percentage of Ter119^+^ cells in bone marrow derived cell preparations) showed a significant shrinkage from 30.20% (control mice) to 19.96% (AIHA mice), i.e. a 33.9% decline. This indicated an overall decline in the erythropoietic activity within the bone marrow of AIHA mice. Enumeration of different erythroid subpopulations in bone marrow showed that each of the individual stages of erythroid differentiation in bone marrow also declined (Fig 7), but only the last stage of erythroid differentiation (erythroblast C) declined more (48.5%) than the overall shrinkage (36.6%) of the erythroid compartment in bone marrow, indicating that erythroblast C was preferentially lost in the bone marrow of AIHA mice. In contrast to the erythropoietic activity in bone marrow, no shrinkage was observed in the erythroid compartment in the spleen as well as in relative proportions of different stages of erythroid differentiation in AIHA spleens. Opposite effects observed in bone marrow and spleen supports the concept of ‘stress erythropoiesis’ [36,37], where a compensatory surge in spleen erythropoiesis sets in when the bone marrow erythropoietic activity suffers a severe decline. It may be noted that the average total recoveries of cells from spleens and bone-marrows of control and AIHA groups of mice were not significantly different. The changes observed in the relative proportions of various subpopulations of cells may therefore be a fair representation of changes in the absolute numbers of cells of these subpopulations.

**Fig 8:**
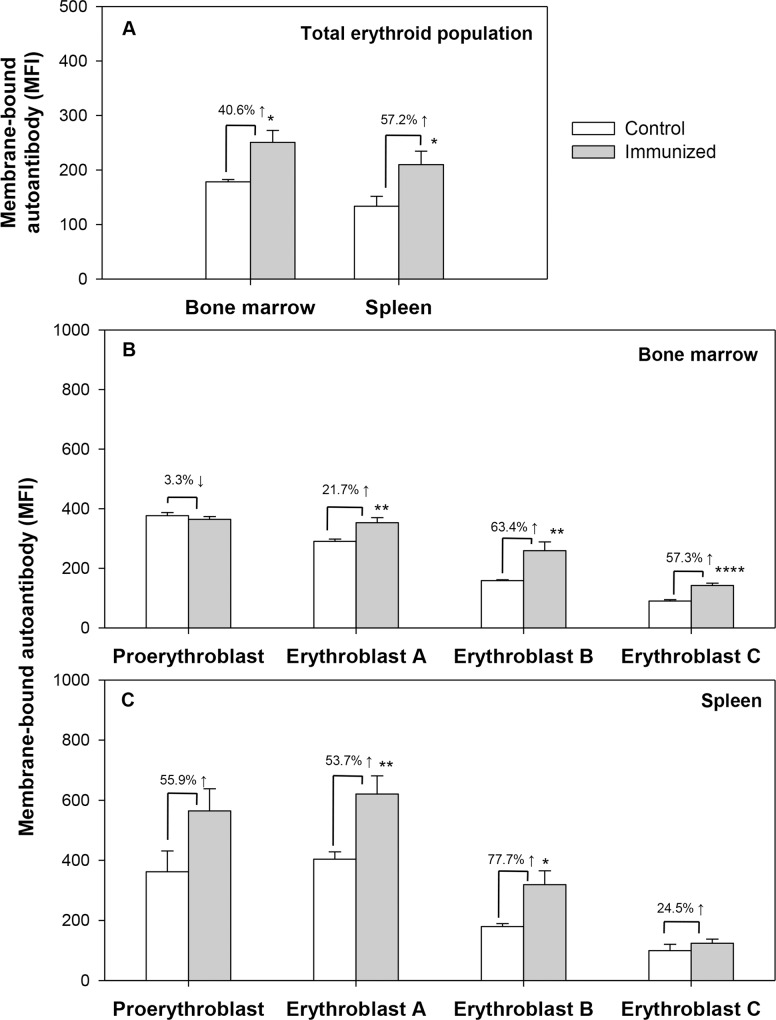
Generation of anti-mouse autoantibody in erythroid cells of bone marrow and spleen in mice with induced AIHA. Mice were given intraperitoneal injections of 2x10^8^ rat erythrocytes weekly for 5–6 weeks to induce AIHA. Mice were sacrificed and their bone marrow and spleen cells harvested. Cells isolated were stained with anti-mouse CD71-PE, anti-mouse Ter119-APC and 7AAD, after blocking with anti-mouse CD16/32, to determine the proportion of live erythroid cells as described in S3 Fig. The erythroid cells were co-stained with F(ab’)_2_ anti-mouse IgG-PE to detect the presence of autoantibodies. Erythroid cells in different stages of maturation (proerythroblasts, erythroblast A, B and C) were gated and autoantibody binding was analyzed in each of them. Presence of membrane-bound autoantibody in the total erythroid populations of bone marrow and spleen after 6 injections is shown in panel A (ANOVA test for bone marrow, *p<*0.001 and spleen, *p =* 0.001). Panels B and C show the binding of autoantibody in erythroid cells at various stages of differentiation in bone marrow and spleen (ANOVA test, *p<*0.001 for both bone marrow and spleen,) respectively. Each bar on the graph represents mean ± SEM of observations. n = 4 control and 6 AIHA-induced mice. *p<0.05, **p<0.01 and ****p<0.001 for comparison of groups (Student t-test).

The presence of membrane-bound autoantibodies on erythroid cells in different stages of differentiation was also examined in bone marrow and spleens of control and AIHA mice. A significant increase in the levels of membrane bound antibodies was observed in the erythroid populations in bone marrow as well as spleens of AIHA mice (Fig 8, panel A). Significant increases in membrane bound antibodies were also seen in cells belonging to individual stages of erythroid differentiation in both bone marrow and spleen (Fig 8, panels B and C). Interestingly, the increase in the level of membrane bound autoantibodies was relatively greater in erythroblast B and erythroblast C populations (63.4% and 57.3% respectively) in bone marrow as compared to the increases seen in the earlier stages of erythroid differentiation.

**Fig 9:**
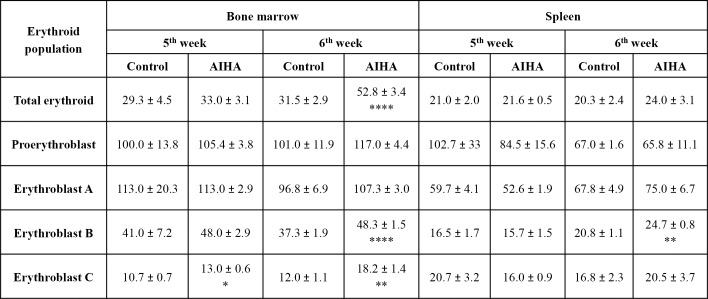
ROS generation in the erythroid cells of bone marrow and spleen in AIHA. Mice were given *i*.*p*. injections of 2x10^8^ rat erythrocytes weekly for 5–6 weeks to induce AIHA. Mice were sacrificed 3 days after 5^th^ and 6^th^ injections and their BM and spleen cells were harvested. Cells isolated were stained with anti-mouse CD71-PE, anti-mouse Ter119-APC and 7AAD, and incubated with CM-H_2_DCFDA. The erythroid cells at different stages of maturation were determined and ROS generation was estimated in each of them. ROS in erythroid cells at different stages of maturation are given above. Each value represents mean ± SEM of observations. n = 4 control and 6 AIHA-induced mice. *p<0.05, **p<0.01 and ****p<0.001 for comparison of the groups (Student t-test).

ROS generation was also examined in cells belonging to different stages of erythroid differentiation in bone marrow and spleen of control and AIHA mice. Results in Fig 9 show a significant increase (67.7%, p<0.001) in ROS levels in all erythroid cells in bone marrow of AIHA mice whereas the magnitude of increase was only 18.5% (not significant) in erythroid cells from spleens of AIHA mice. Comparison of ROS levels in different stages of bone marrow erythroid cells showed that significant increase in ROS levels were observed only for erythroblast B and erythroblast C stages and within these two stages, maximum increase (51.4%) in ROS levels was seen in erythroblast C stage (Fig 9), that also showed the maximum decline in proportions in AIHA mice (Fig 7).

## Discussion

Autoimmune diseases are brought about by a breakdown in self tolerance, caused by activation of self reactive lymphocytes [38]. Immunological unresponsiveness is attained via clonal deletion [39,40] and/or clonal anergy [40,41] of lymphocytes that may potentially react with self-components. Self tolerance can be broken in certain cases due to genetic and/or environmental factors such as infections [42,43] that may provoke autoimmunity by either exposure of cryptic self epitopes or neoantigens, polyclonal T- and/or B-cell activation, molecular mimicry between self and foreign antigens, errors in central and peripheral tolerance, or due to disorders of immune regulation [44]. The Playfair and Clarke model [20] of experimentally induced AIHA in mice employs one such mechanism, where the immunological unresponsiveness to self erythrocytes is broken by repeated exposure to rat erythrocyte antigens with similar/shared epitopes that activate the otherwise inactive autoreactive B- and T-lymphocytes [22–23]. The rat erythrocyte administered (REA) mice develops autoimmune haemolytic anemia (AIHA) characterized by the presence of anti-mouse erythrocyte autoantibody [22].

In our study, C57BL/6 mice developed significant anemia after 5–6 consecutive weekly injections (*i*.*p*.) of rat erythrocytes along with significant levels of bound autoantibody levels on erythrocytes and erythroid cells in bone marrow and spleen. A detailed study involving the subpopulations of erythrocytes of different age groups was undertaken to analyze the age-dependent susceptibility of circulating erythrocytes to removal during AIHA. Our study revealed a significant decline in the relative proportion of younger erythrocytes and a concomitant significant increase in the relative proportions of old erythrocytes in circulation (Fig 3, panels D-G). These results are indicative of a preferential loss of younger erythrocytes and an accumulation of old erythrocytes in the circulation of AIHA mice. Interestingly, the membrane bound autoantibody could be demonstrated on all age groups of erythrocytes, especially after 5 weekly injections of rat erythrocytes. At sixth week time point, significant erythrocyte-bound autoantibody was still demonstrable for reticulocytes and young erythrocytes. Autoantibody binding was also seen in all stages of erythroid differentiation in bone marrow as well as in spleen. Levels of these membrane bound autoantibodies are however maximum in the Erythroblast C stage in bone marrow and in reticulocytes and the youngest erythrocytes in circulation. Since membrane bound antibodies may induce cell elimination through normal mechanisms of complement activation and antibody mediated phagocytosis, these mechanisms may contribute to a greater susceptibility of these stages in the life cycle of erythroid cells to elimination in AIHA. Another factor that may render cells more susceptible to elimination could be the generation of ROS in response to autoantibodies. Presence of autoantibody on bone marrow progenitor cells has been linked to the development of hypoplasia, and even pure red cell aplasia in Systemic lupus erythematosus [36,37]. Like the levels of membrane bound autoantibodies, Erythroblast C stage in bone marrow and reticulocytes and young erythrocytes in blood circulation generate higher levels of ROS and this factor too may contribute to the preferential elimination of these cells in AIHA.

Binding of autoantibodies may promote the lysis of erythrocytes by compliment activation as well as through opsonization resulting in enhanced phagocytosis. Both these processes may however require a certain critical density of autoantibodies on erythrocyte membrane and autoantibody response being a poor one, may not always result in erythrocytes having the required critical levels of membrane bound antibody. Accordingly, not all erythroid cells and erythrocyte sub-populations that had bound autoantibody, declined in relative proportions. Antibody binding may also induce other changes in erythrocytes that may influence the survival of the cells. Autoimmune response has been linked to ROS-mediated damage in a variety of autoimmune diseases [31–35]. Autoantibodies to antioxidant enzymes have been reported that induce oxidative stress, which in turn leads to the generation of oxidatively modified autoantigens that serve as neoantigens, eliciting more inflammatory response [33,45]. In AIHA, generation of autoantibodies against highly antigenic band 3 protein have been demonstrated [23], that bind anion channel and block the release of intracellular ROS, particularly superoxide anion from erythrocytes, resulting in increased intracellular ROS levels [34,35]. ROS generated in erythrocytes could therefore be a contributory factor in determining the susceptibilities of different age cohorts of erythrocytes to elimination. Our study revealed a significant increase (34.6%) in the ROS level in whole blood erythrocytes as well as all the age defined subpopulations of erythrocytes in AIHA (Fig 5, panel B-F). Maximum ROS level was however recorded in the young biotin^negative^ erythrocytes (Fig 5, panel D) which could be a factor in increased susceptibility of this group of erythrocytes to elimination mechanisms. Old erythrocytes, in spite of binding autoantibodies and elevated ROS levels, seem to survive in AIHA for which the reason is not clear. It is possible that the decreased susceptibility of old erythrocytes may result from decreased efficiency of cell death or eryptosis inducing mechanisms that binding of autoantibodies may induce. Verification of this proposition would need further work.

**Fig 10:**
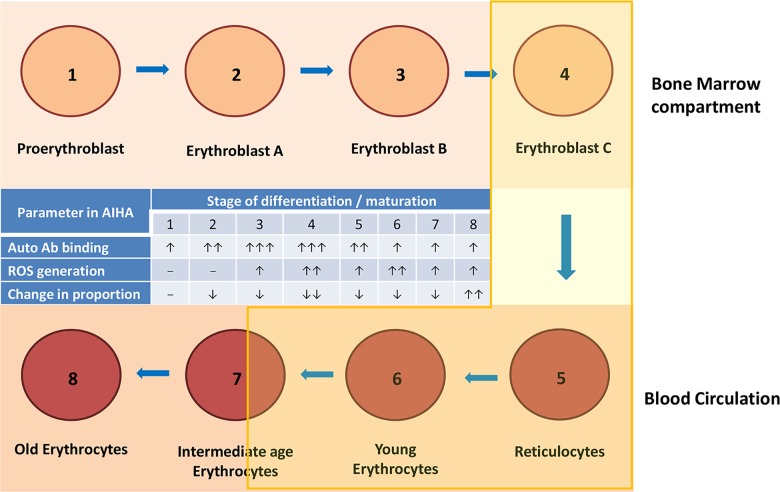
Proposed model of AIHA. A model to depict the stages in the life cycle of erythroid cells that are preferentially eliminated in AIHA is given above. Stages 1 to 4 depict erythroid stages in bone marrow and stages 5–8 depict different age groups of erythrocytes in blood circulation. A summary of results on binding of autoantibodies and ROS generation in different stages and changes in relative proportions of cells in various stages are shown in the table within the figure. Shaded area covering stages 4 till 7 are preferentially eliminated in AIHA mouse model.

Based upon our results, we would like to propose a model to define the susceptibility of erythroid cells undergoing differentiation and age defined subpopulations of blood erythrocytes (Fig 10). In this model, eight stages of lifecycle of erythroid cells have been shown out of which first four are in bone marrow and rest in blood circulation. Trends in binding of the autoantibody generated in AIHA mice as well as relative generation of ROS in different stages are depicted in the table provided within the illustration of the model. Changes in the relative proportions of these stages in the life cycle of erythroid cells within bone marrow (stages 1–4) and in blood (stages 5–8) are also compared in this table. Within bone marrow, the proportion of stage 4 alone falls significantly in relation to a general contraction of the erythroid population in bone marrow of AIHA mice. In blood circulation stages 5–7 fall significantly whereas the proportion of stage 8 (old erythrocytes) actually increases significantly. Thus there seem to be a preferential elimination of stages 4 in bone marrow and stages 5–7 in blood in AIHA even though the binding of autoantibodies and ROS generation may also be seen in other stages. It appears therefore that in AIHA, cells in early erythroid differentiation stages as well as the old erythrocytes in blood circulation are not much affected. Stage 4 in bone marrow and 5–7 in blood erythrocytes seem to be preferentially eliminated in AIHA. Our results thus indicate that in the AIHA mouse model, later stages of bone marrow erythroid differentiation and younger erythrocytes in blood circulation are specifically eliminated. This is new information and may help in designing appropriate interventions for AIHA.

## Supporting Information

S1 Fig Double *in vivo* biotinylation (DIB) technique for tracking age related changes on circulating erythrocytes. C57BL/6 mice were administered three daily (*i*.*v*.) doses of 1 mg BXN (first biotinylation step). After a rest for 30 days, a single additional dose of 0.6 mg BXN was administered (second biotinylation step). Blood was collected at different time points and distribution of biotin label on erythrocytes was examined by staining the cells with streptavidin-APC followed by flow cytometry. Biotin^negative^ erythrocytes would represent fresh and youngest erythrocytes released in blood after the second biotinylation step, biotin^low^ erythrocytes, the cohort of erythrocytes released in circulation between the first and the second biotinylation steps, and biotin^high^ erythrocytes would represent the population of old residual erythrocytes that were present in blood at the time of first biotinylation step. Young erythrocyte group could further be subdivided into reticulocytes and young erythrocytes based upon staining with CD71 antibody. The scheme of the experiment is given in panel A, and the gating strategy used for the identification of biotin^high^, biotin^low^, CD71^˗^biotin^negative^ erythrocytes and CD71^+^biotin^negative^ reticulocytes following flow cytometry is given in panel B.
(TIF)

S2 Fig Generation of anti-mouse erythrocyte autoantibody in mice with induced AIHA. C57BL/6 mice were given *i*.*p*. injections of 2x10^8^ rat erythrocytes weekly for 5–6 weeks to induce AIHA. At intended time points mice were bled and erythrocytes (1x10^6^) were stained with anti-mouse IgG/IgM-FITC polyclonal antibody to assess the presence of membrane-bound autoantibody in erythrocytes in control and AIHA-induced mice. Representative flow histograms showing anti-mouse IgG/IgM-FITC staining is given in panel A. The level of membrane-bound autoantibody on circulating erythrocytes is given in panels B (relative binding of autoantibody) and C (proportion of erythrocytes with membrane-bound autoantibody). Each bar in the graph represents mean ± SEM of observations. n = 10 mice. ***p<0.005 and ****p<0.001 for comparison of the groups (Student t-test).
(TIF)

S3 Fig Erythropoietic activity in the bone marrow (BM) and spleen in mice with induced AIHA. Mice were given intraperitoneal injections of 2x10^8^ rat erythrocytes weekly for 5–6 weeks to induce AIHA. Mice were sacrificed 3 days after 5^th^ and 6^th^ doses of injection and their bone marrow and spleen cells were harvested. Cells isolated were stained with anti-mouse CD71-PE, anti-mouse Ter119-APC and 7AAD, after blocking with anti-mouse CD16/32, and the proportions of erythroid cells were determined. The gating strategy for determining the erythroid cells at different stages of maturation is shown above. Briefly the bone marrow and spleen cells were gated as live 7AAD^-^ population (panel B) and delineated as per CD71 and Ter119 levels. Ter119^+^ erythroid cells could be identified within an inverted ‘L’ shaped gate in the flow diagram (panel C). The Ter119^med^CD71^high^ erythroid cells within this inverted ‘L’ can be identified as the early proerythroblasts (panel C). The remaining erythroid population can be further delineated into three different populations as based on their size (Forward Scatter, FSC) and CD71 staining (panel D). These include early basophilic erythroblasts or erythroblasts A (Ter119^high^CD71^high^FSC^high^), late basophilic polychromatic and orthochromatic erythroblasts or erythroblasts B (Ter119^high^ CD71^med^FSC^low^), and orthochromatic erythroblasts with mature erythrocytes or erythroblasts C (Ter119^high^CD71^low^FSC^low^).
(TIF)
