# Supplementary figures and images for: Identification of Stages of Erythroid Differentiation in Bone Marrow and Erythrocyte Subpopulations in Blood Circulation that Are Preferentially Lost in Autoimmune Hemolytic Anemia in Mouse
Source: PLoS One. 2016 Nov 21;11(11):e0166878. doi: 10.1371/journal.pone.0166878 (PMC5117735; doi:10.1371/journal.pone.0166878)

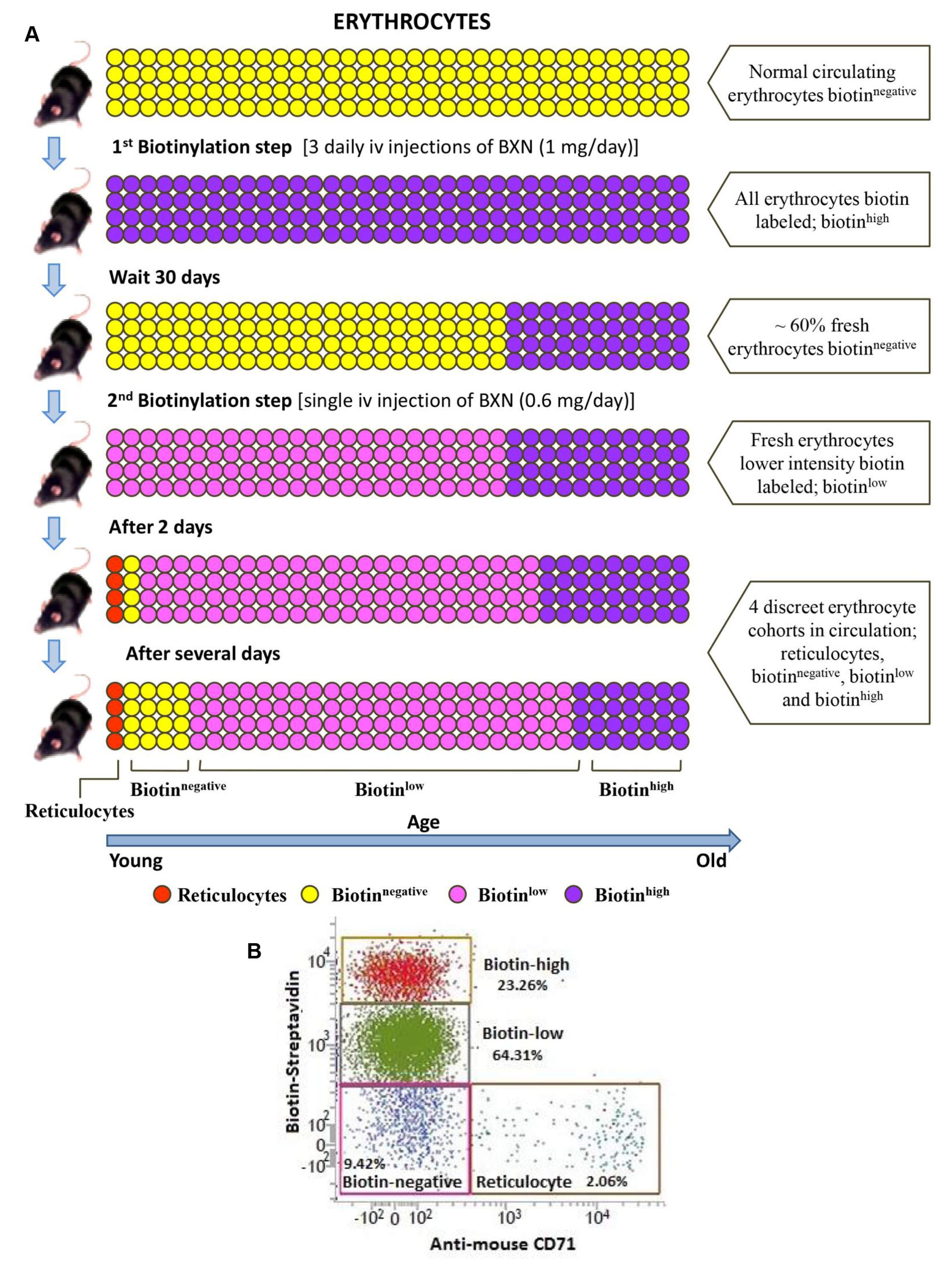

Supplement: S1 Fig — C57BL/6 mice were administered three daily (i.v.) doses of 1 mg BXN (first biotinylation step). After a rest for 30 days, a single additional dose of 0.6 mg BXN was administered (second biotinylation step). Blood was collected at different time points and distribution of biotin label on erythrocytes was examined by staining the cells with streptavidin-APC followed by flow cytometry. Biotinnegative erythrocytes would represent fresh and youngest erythrocytes released in blood after the second biotinylation step, biotinlow erythrocytes, the cohort of erythrocytes released in circulation between the first and the second biotinylation steps, and biotinhigh erythrocytes would represent the population of old residual erythrocytes that were present in blood at the time of first biotinylation step. Young erythrocyte group could further be subdivided into reticulocytes and young erythrocytes based upon staining with CD71 antibody. The scheme of the experiment is given in panel A, and the gating strategy used for the identification of biotinhigh, biotinlow, CD71˗biotinnegative erythrocytes and CD71+biotinnegative reticulocytes following flow cytometry is given in panel B. (TIF) [file pone.0166878.s001.tif]

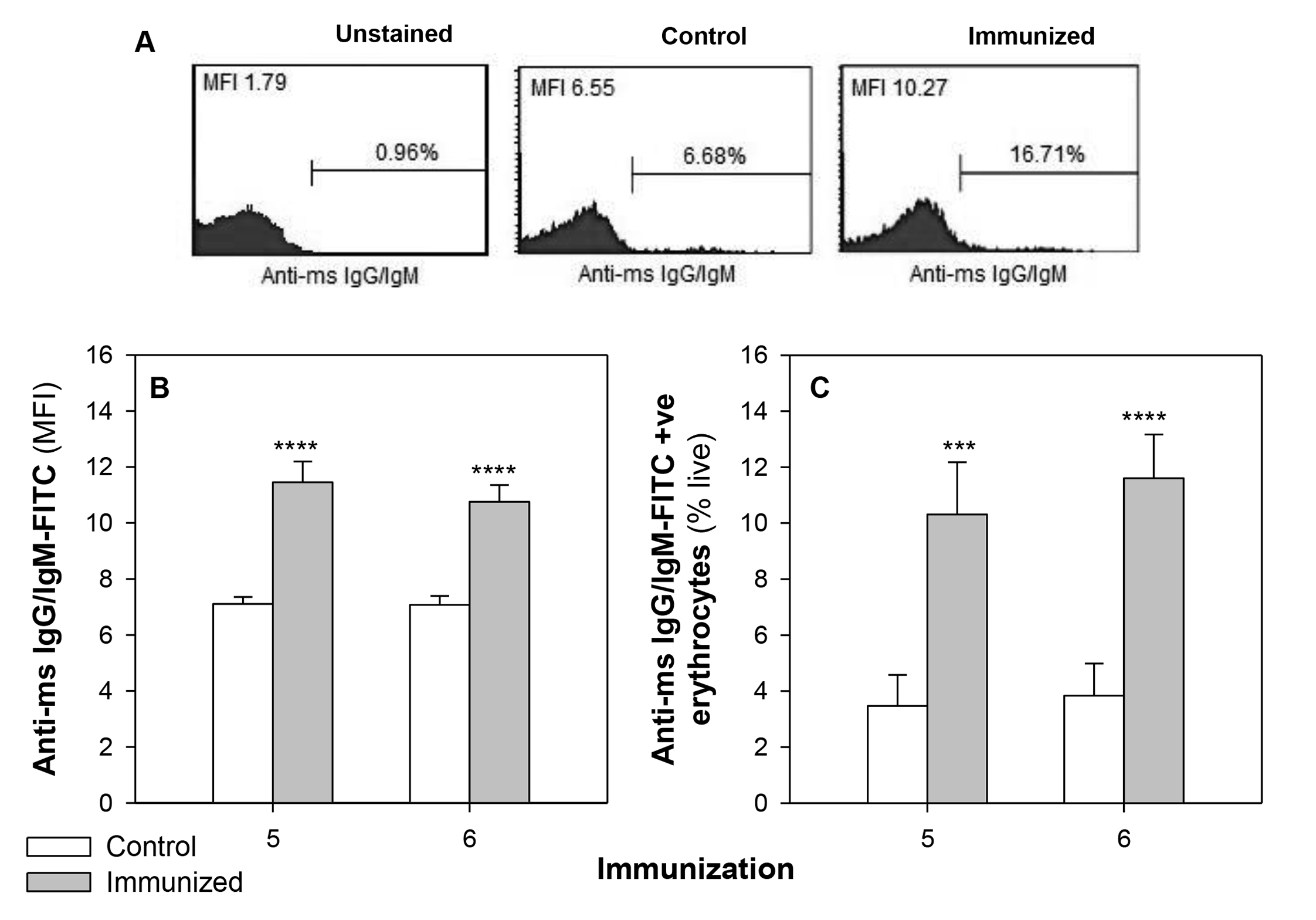

Supplement: S2 Fig — C57BL/6 mice were given i.p. injections of 2x10^8^ rat erythrocytes weekly for 5–6 weeks to induce AIHA. At intended time points mice were bled and erythrocytes (1x10^6^) were stained with anti-mouse IgG/IgM-FITC polyclonal antibody to assess the presence of membrane-bound autoantibody in erythrocytes in control and AIHA-induced mice. Representative flow histograms showing anti-mouse IgG/IgM-FITC staining is given in panel A. The level of membrane-bound autoantibody on circulating erythrocytes is given in panels B (relative binding of autoantibody) and C (proportion of erythrocytes with membrane-bound autoantibody). Each bar in the graph represents mean ± SEM of observations. n = 10 mice. ***p<0.005 and ****p<0.001 for comparison of the groups (Student t-test). (TIF) [file pone.0166878.s002.tif]

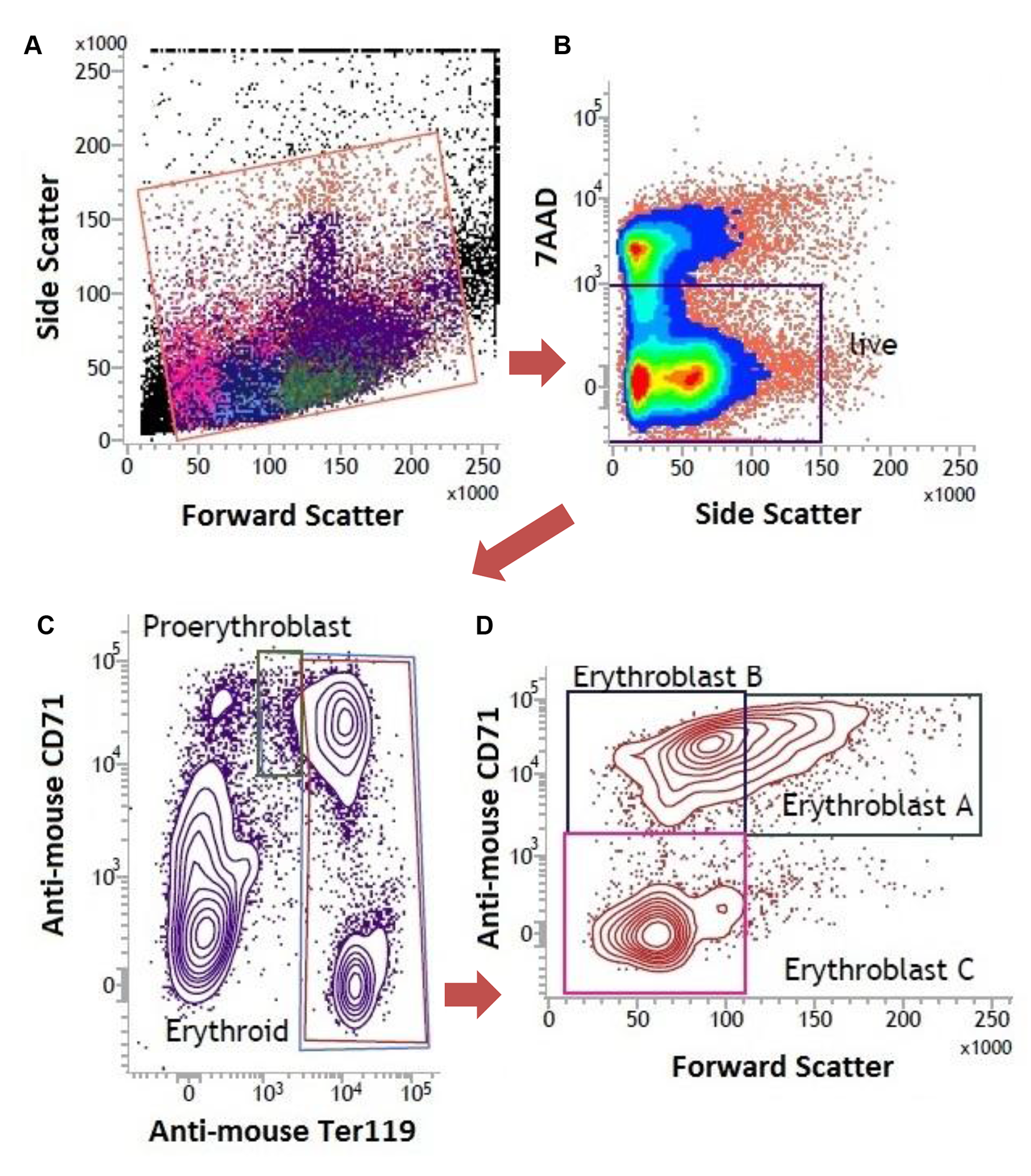

Supplement: S3 Fig — Mice were given intraperitoneal injections of 2x10^8^ rat erythrocytes weekly for 5–6 weeks to induce AIHA. Mice were sacrificed 3 days after 5th and 6th doses of injection and their bone marrow and spleen cells were harvested. Cells isolated were stained with anti-mouse CD71-PE, anti-mouse Ter119-APC and 7AAD, after blocking with anti-mouse CD16/32, and the proportions of erythroid cells were determined. The gating strategy for determining the erythroid cells at different stages of maturation is shown above. Briefly the bone marrow and spleen cells were gated as live 7AAD- population (panel B) and delineated as per CD71 and Ter119 levels. Ter119+ erythroid cells could be identified within an inverted ‘L’ shaped gate in the flow diagram (panel C). The Ter119medCD71high erythroid cells within this inverted ‘L’ can be identified as the early proerythroblasts (panel C). The remaining erythroid population can be further delineated into three different populations as based on their size (Forward Scatter, FSC) and CD71 staining (panel D). These include early basophilic erythroblasts or erythroblasts A (Ter119highCD71highFSChigh), late basophilic polychromatic and orthochromatic erythroblasts or erythroblasts B (Ter119high CD71medFSClow), and orthochromatic erythroblasts with mature erythrocytes or erythroblasts C (Ter119highCD71lowFSClow). (TIF) [file pone.0166878.s003.tif]
